# Supplementary material for: Stable Integration of Inducible SPLICS Reporters Enables Spatio-Temporal Analysis of Multiple Organelle Contact Sites upon Modulation of Cholesterol Traffic
Source: Cells. 2022 May 14;11(10):1643. doi: 10.3390/cells11101643 (PMC9139547; doi:10.3390/cells11101643)
Supplement: Supplementary file 1 [file cells-11-01643-s001.zip › cells-1713868-supplementary.pdf]

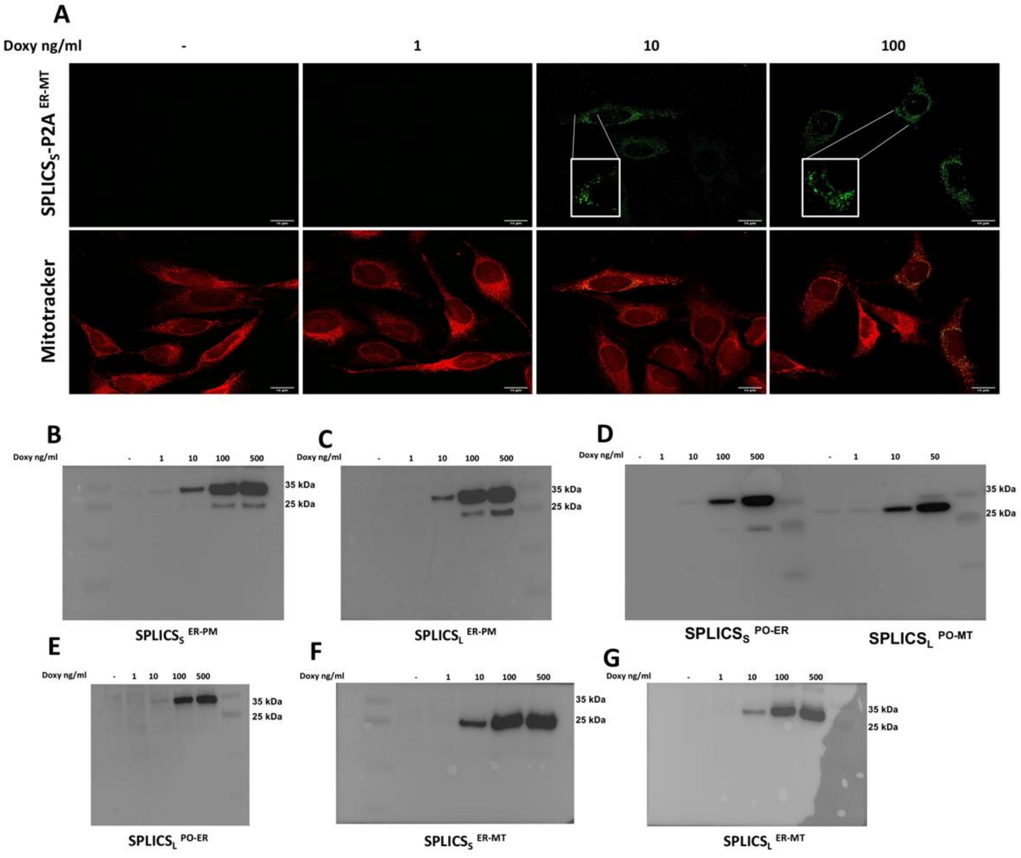

Figure S1. Characterization of Hela polyclonal stable cell lines expressing SPLICS$_{S/L}$-P2A^{ER-PM} and PO-ER, SPLICS$_{S/L}$-P2A^{ER-MT} and SPLIC$_{S/L}$-P2A^{PO-MT}. (A) Representative full-scan image reporting the typically dot pattern of ER-mitochondria interaction detected in Hela polyclonal stable cell lines expressing SPLICS$_S$-P2A^{ER-MT} treated with increasing concentration of doxycycline after 8 hours induction. (B-H) Representative membrane for western blotting analysis of Hela polyclonal stable cell lines lysates expressing SPLICS$_{S/L}$-P2A probes and stimulated with increasing concentration of doxycycline for 24h. GFP portions have been detected with an anti-GFP antibodies and an equal amount of total loaded lysate was verified by incubation with anti-β Tubulin antibody.

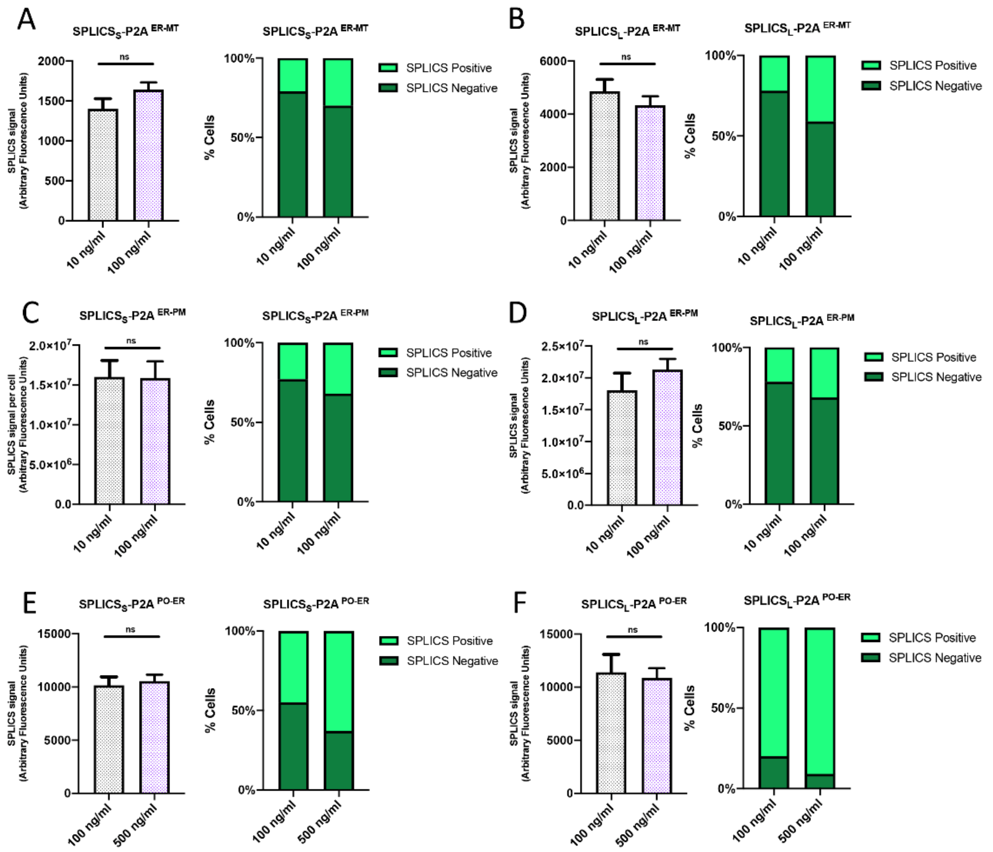

Figure S2. Characterization of Hela polyclonal stable cell lines expressing SPLICS$_{S/L}$$^{ER-MT}$, SPLICS$_{S/L}$-P2A^{ER-PM} and SPLICS$_{S/L}$-P2A^{PO-ER}. (A-B) Quantification of the SPLICS fluorescence intensity and % of SPLICS expressing cells at 10 and 100 ng/ml of doxycycline for SPLICS$_{S/L}$-P2A^{ER-MT}. (C-D) Quantification of the SPLICS fluorescence intensity and % of SPLICS expressing cells at 10 and 100 ng/ml of doxycycline for SPLICS$_{S/L}$-P2A^{ER-PM}. (E-F) Quantification of the SPLICS fluorescence intensity and % of SPLICS expressing cells at 100 and 500 ng/ml of doxycycline for SPLICS$_{S/L}$-P2A^{PO-ER}.

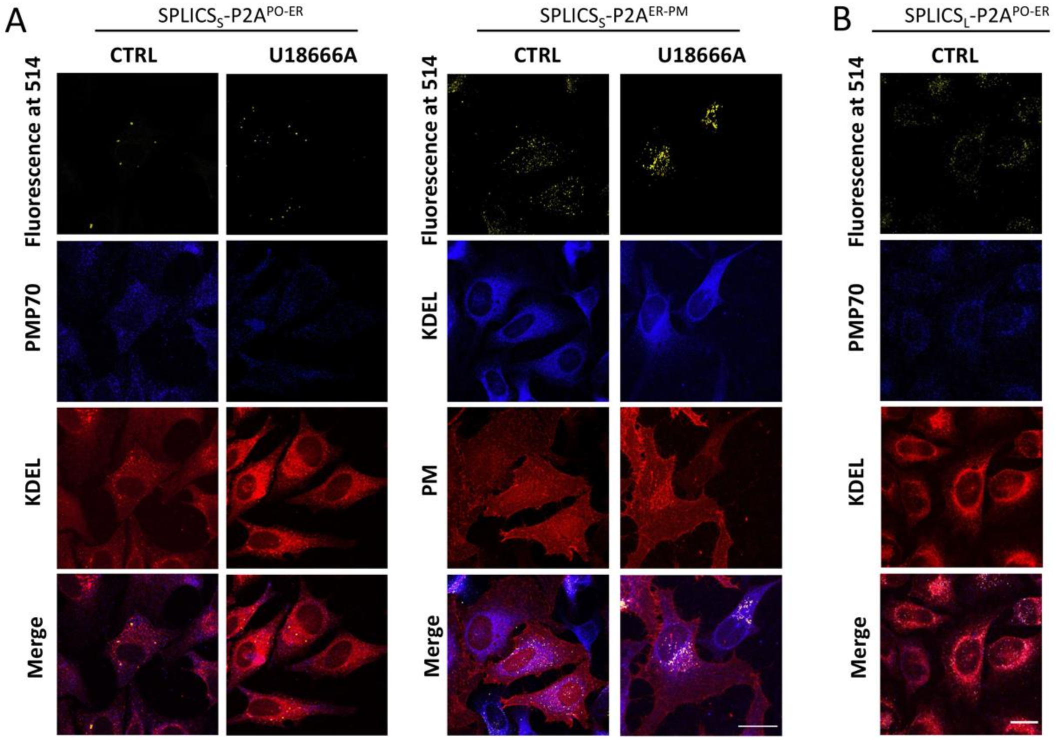

Figure S3. Immunofluorescence analysis in Hela polyclonal stable cell lines expressing SPLICS$_S$-P2A^{ER-PM}, SPLICS$_S$-P2A^{PO-ER}. (A) Immunofluorescence analysis in cells untreated and treated with U18666A showing the morphology of ER and peroxisomes in Hela polyclonal stable cell lines expressing SPLICS$_S$-P2A^{PO-ER}, and the morphology of ER and plasma membrane (PM) in Hela polyclonal stable cell lines expressing SPLICS$_S$-P2A^{ER-PM}. Scale bar: 20 μm. (B) Immunofluorescence analysis in cells untreated showing the morphology of ER and peroxisomes in Hela polyclonal stable cell lines expressing SPLICS$_L$-P2A^{PO-ER}. Scale bar: 20 μm.
